# Technologies for Advanced Gait and Balance Assessments in People with Multiple Sclerosis

**DOI:** 10.3389/fneur.2017.00708

**Published:** 2018-02-02

**Authors:** Camille J. Shanahan, Frederique M. C. Boonstra, L. Eduardo Cofré Lizama, Myrte Strik, Bradford A. Moffat, Fary Khan, Trevor J. Kilpatrick, Anneke van der Walt, Mary P. Galea, Scott C. Kolbe

**Affiliations:** ^1^Department of Anatomy and Neuroscience, University of Melbourne, Parkville, VIC, Australia; ^2^Department of Medicine, University of Melbourne, Parkville, VIC, Australia; ^3^Australian Rehabilitation Research Centre, Royal Melbourne Hospital, Parkville, VIC, Australia; ^4^Department of Anatomy and Neuroscience, VU Medical Centre, Amsterdam, Netherlands; ^5^Florey Institute of Neuroscience and Mental Health, Parkville, VIC, Australia

**Keywords:** multiple sclerosis, mobility loss, gait, balance, biomechanics

## Abstract

Subtle gait and balance dysfunction is a precursor to loss of mobility in multiple sclerosis (MS). Biomechanical assessments using advanced gait and balance analysis technologies can identify these subtle changes and could be used to predict mobility loss early in the disease. This update critically evaluates advanced gait and balance analysis technologies and their applicability to identifying early lower limb dysfunction in people with MS. Non-wearable (motion capture systems, force platforms, and sensor-embedded walkways) and wearable (pressure and inertial sensors) biomechanical analysis systems have been developed to provide quantitative gait and balance assessments. Non-wearable systems are highly accurate, reliable and provide detailed outcomes, but require cumbersome and expensive equipment. Wearable systems provide less detail but can be used in community settings and can provide real-time feedback to patients and clinicians. Biomechanical analysis using advanced gait and balance analysis technologies can identify changes in gait and balance in early MS and consequently have the potential to significantly improve monitoring of mobility changes in MS.

## Introduction

Mobility loss in people with multiple sclerosis (pwMS) is a major contributor to decreased quality of life, disruption to employment, and increased financial burden (1, 2). Motor weakness, loss of coordination, and spasticity can all manifest canonically as alterations in walking (gait) and balance that ultimately lead to mobility loss. Subtle gait and balance changes are apparent in pwMS even at the earliest disease stages and can be measured using advanced movement analysis techniques (3–5). Given their sensitivity, advanced movement analysis techniques could be used to identify patients at risk of mobility loss (6) or as outcomes in trials of therapies to preserve mobility.

Clinical assessment of gait in pwMS often involves visual evaluation and walking performance, tests of maximum distance walked, or timed walks (7). Both visual and performance tests are relatively reliable over time (8, 9); however, reliability varies with the degree of disability (8–10), and the tests are insensitive to subtle changes early in the disease (3–5, 7, 11).

Over the past two decades, advanced movement analysis technologies have been developed to improve objectivity, accuracy, quantification, and sensitivity to disease-related changes of clinical assessments of gait and balance (12–14). Advanced movement analysis technologies measure aspects of lower limb functions such as positions, angles, velocities, accelerations (kinematics), and forces and moments (kinetics) of limb segments and joints during walking. As such, these technologies can provide more sensitive markers of changes in walking and balance in pwMS than standard clinical assessments.

This review aims to present a synopsis of techniques that we consider to have potential utility for gait and balance assessment in pwMS and a discussion of the techniques when applied to gait/balance assessment in pwMS. We review both non-wearable and wearable gait analysis systems and discuss the variables measured by these systems as well as advantages, disadvantages, sensitivity, and accuracy. This information is also summarized in Table 1 for reference.

**Table 1:** Comparison of advanced techniques used for gait assessment in people with multiple sclerosis (MS).

Assessment technique	Outcome measures	Advantages	Disadvantages	Accuracy/reliability	Application in MS
Marker-based motion capture	Spatial and temporal variables Kinematics	Comprehensive analysis of widest range of gait variables Power consumption is not an issue Little interferences from external environmental factors	Expensive Must be used in a laboratory environment Markers and restricted space can hinder movement	Reliability between trials (ICC) = 0.95–1.00 (15)	GRFs, temporal-spatial measures and ankle, knee, and hip angles throughout gait differ between mild MS patients and controls (3) Spatiotemporal variables and ankle, knee, and hip angles differ in people with MS compared to controls and differences are more pronounced with increasing disease severity (4, 16) Change in balance measures contributes to deficits in walking performance over time in patients with established MS (17) Slower preferred walking speeds with longer dual support; dual support times were longer and swing times were shorter even at fixed walking speeds (18)
Markerless motion capture	Spatial and temporal variables Kinematics	Objectivity Quantification High sensitivity Comprehensivene Better suited to clinical environments than marker-based systems	Can be expensive Generally cannot be used outside the clinic or laboratory environment Measure a restricted number of steps	ToF: accuracy = 84–94% (19) Kinect: <1% mean error compared to marker-based (20) Reliability (ICC) = 0.91–0.98 (15)	ToF used to provide video-based rehabilitation to increase motivation and treatment efficacy for people with MS. Usability and benefits highly rated. System supports rehabilitation by allowing for real-time correction of abnormal movements (21) Kinect can detect differences in gait speed and gait “left/right deviation” in people with MS compared to controls, and results correlate with EDSS and T25FW scores (22)
Force platforms	GRF pattern Kinematics	Objectivity Quantification Good sensitivity	Restricted to laboratory environments	Reliability (ICC) = 0.22–0.97 (23) CoP error = 1.8 mm Orientation error = 1.0% (24) Treadmill mounted force platforms simple gait variables are high (ICC = 0.86–0.97); for gait variability the reliability is low to moderate (ICC = 0.22–0.44) (23)	Changes in walking and jogging gait variables in people with MS with minimal disability compared to controls, with greater change found during jogging compared to walking (25)
Wii Balance Board	GRF pattern	Objectivity Quantification Portability	Clinical, research and home	Excellent ICCs. Test–retest reliability (0.66–0.94), construct validity (0.77–0.89) (26, 27)	Wii Balance Board can discriminate fallers and non-fallers with MS (28) In a single case study Wii Balance Board Measure could predict relapse onset and assess intervention efficacy (29)
Instrumented walkways (GAITRite)	Spatial and temporal variables	Clinical feasibility Objectivity Quantification Good sensitivity	Restricted to clinic or laboratory environments Restricted to few steps at a time	MDC = 7–20% (in older adults) (30) Reliability (ICC) = 0.69–0.99 (31) 1.5% mean error compared to motion capture (32)	Quantitative spatiotemporal gait variables (33, 34) Sensitive in patients with minimal disability (35) Similar clinical validity as T25FW in people with MS (36) Detects changes in gait in very early-stage MS patients with minimal disability (35, 37) Gait variables correlate with EDSS system domains (38)
Pressure sensors	Spatial and temporal variables	Clinical feasibility Objectivity Quantification Good sensitivity Can be used outside the clinic and laboratory	Sensors can impede movement Battery powered	Reliability (ICC) = 0.90–0.99 (39) Correlation with motion capture > 0.95 Mean error < 5.4% compared to motion capture (40)	Differences in gait variability and sites of foot pressure throughout gait cycle between MS patients and controls (41)
Inertial sensors	Spatial and temporal variables Kinematics	Clinical feasibility Objectivity Quantification Good sensitivity Face validity	Sensors can impede movement Battery powered Susceptible to environmental interference May need technical operators	Mean error < 5% compared to motion capture (42) Detection accuracy > 80% (43) Reliability (ICC) = 0.90–0.99 (44)	Can detect changes balance, gait dysfunction, and arm movement during walking otherwise undetected by timed walking tests in MS patients with minimal disability (45, 46) Capable of separating mild MS (average EDSS = 2.2), moderate MS (average EDSS = 4.3) and controls based on gait velocity, trunk motion, sway range, and sway area (14)

*MDC, minimal detectable difference; ICC, intraclass correlation coefficient; CoP, center of pressure; ToF, time of flight; GRF, ground reaction force*.

## Non-Wearable Gait Analysis Technologies

Non-wearable technologies generally provide the most sensitive and accurate gait data, yet require dedicated laboratory environments and are expensive compared to wearable systems (47). Three main non-wearable technologies are as follows: optical motion capture systems, force platforms/balance boards, and instrumented walkway mats.

### Optical Motion Capture

Optical motion capture systems are based on optoelectronic stereophotogrammetry and measure kinematics of gait in three dimensions (47–50). These systems include marker-based and marker-less systems.

#### Marker-Based Systems

Marker-based systems utilize reflective markers placed on anatomical landmarks (e.g., joints) allowing them to capture motion of body parts. These systems are highly accurate (mean noise estimate = 0.03–0.05%) and reproducible [intraclass correlation coefficient (ICC) > 0.95] (15, 51). These systems can track the whole body, allowing them to record the most extensive range of kinematic variables of any gait assessment technique. These systems can be combined with force plates and/or electromyography (EMG) to collect ground reaction force (GRF) and muscle activation, enabling simultaneous assessments of kinematics and forces. The key limitation of marker-based systems is the need for dedicated spaces and technical operators, making them expensive and of limited clinical utility.

Several studies have used marker-based systems to quantify kinematic changes in gait and balance in pwMS (3, 4, 16, 18, 52). These studies show that, compared to healthy controls, pwMS displays: (1) reduced gait speed and stride length and prolonged double support time, even with fixed walking speed (3, 4, 16, 18), (2) differences in hip, knee, and ankle motion (3, 4, 16), and (3) abnormal timing of tibialis anterior and gastrocnemius activation (3, 4) with the degree of gait impairment associated with disease severity (4, 16). Reduced stride length appeared to be a consequence of reduced hip extension in mid and terminal stance, together with knee extension in late swing and at heel strike (16). Although increased double support time is usually interpreted as a strategy for increasing stability during gait, the opposite is true if destabilizing swing dynamics exist, particularly at non-preferred walking speeds (18). This could in part explain concomitant alterations to head and body centers of mass positions throughout gait that could provide additional stability (18). Indeed, two studies by Peebles and colleagues noted that dynamic stability (measured as the margin of stability which relates to the motion of the center of mass relative to the foot strike) worsened at faster walking speeds in pwMS and clinical gait disturbance (53) and was more severe in patients with a history of falls (54).

Two longitudinal studies have studied changes in gait using marker-based systems (17, 52). Fritz et al. (17) found no significant change in gait velocity over 2–3 years in 57 pwMS, despite an increase in T25FW. However, the authors did not provide a comprehensive assessment of gait function (e.g., timing of gait cycle events or joint motion), potentially limiting their ability to detect subtle changes. Galea et al. (52) noted a range of progressive changes over a brief 12-month period in 38 pwMS and mild diseases (EDSS < 3) including changes in ankle kinematics.

#### Marker-Less Systems

Although not as accurate and reliable as marker-based optical motion capture, marker-less motion tracking has the advantages of reduced preparation time and no hindrance to movement by body-mounted markers. Two categories of marker-less motion capture systems are available: active and passive vision systems. Active systems emit visible or infrared light using either laser, patterned or modulated light pulses. Passive systems utilize real-time image analysis.

Time of flight (ToF) systems are active marker-less systems that measure the motion of joints and segments across the whole body. ToF systems emit light (often infrared) that is reflected by all objects in the scene. A sensor is used to capture the reflected light and to calculate the distance based on the phase shift between the emitted and reflected light (55). These systems use self-contained light sources and a single camera making them relatively cheap and robust to differences in illumination. Recent advances in ToF systems have increased the accuracy of identification of gait patterns to 84–94% (19); however, the reliability of ToF has not been established. A single pilot study in pwMS employed ToF-based video applications during patient rehabilitation to improve usability and increase motivation (21). The real-time feedback from ToF allowed patients to self-correct abnormal movements, which was seen as a positive feature (21).

Similar to ToF, structured light systems operate by analyzing the deformation of a reflected light beam. The Kinect^®^ sensor developed for video gaming is one of the most commonly used structured light systems due to its low cost (20, 35). Kinect can measure spatiotemporal features of gait such as heel strike and toe off, as well as knee and hip angles (56). Algorithms have been developed to improve the accuracy of gait measurements with Kinect, resulting in mean error estimates of <1% (20, 35, 57). Several studies have demonstrated that Kinect can accurately assess stride dynamics during walking to provide measures of walking speed, stride time, and stride length in healthy subjects (20, 35, 58, 59). Kinect has been used in a single study of MS patients during T25FW (22 MS patients, median EDSS = 3) (22). The investigators found differences in the degree of directional variability of gait, with good test–retest reliability (ICC > 0.9). Gait speed measured with Kinect correlated with T25FW time and EDSS (including brainstem and pyramidal subscores) (22). Further investigations are required to determine the applicability and reliability of Kinect for gait analysis in larger MS cohorts in clinical and home environments.

### Force Platforms

Force platforms are steel blocks equipped with strain gauges or piezoelectric transducers measure GRF and can be embedded in a walkway or in treadmills for continuous recordings of multiple gait cycles. The gait cycle results in a repetitive and unique GRF pattern with precisely timed events such as heel-contact and toe-off that can be quantitatively assessed (60). Additionally, center of pressure (CoP) can be measured continuously between the body and ground as an indicator of balance. Force platforms are generally expensive and require dedicated laboratory environments and skilled technical personnel to operate. However, they can be used in conjunction with motion capture and EMG systems to provide joint kinetics (moments, power, and forces applied by each joint when braking or propelling) making them useful for laboratory-based assessments of gait and balance in pwMS. Additionally, graphical representations of gait, known as “butterfly diagrams,” can be produced that represent the 2D envelop of the GRF vectors during a step and could have clinical utility (25).

In-floor force platforms display high test–retest reliability for gait (61) and balance (62–65) variables. The reliability of treadmill-based force platforms for simple gait variables (mean stride frequency, stride width, time and length, and double stance phase) is also high (ICC = 0.86–0.97); however, for more complex measures such as gait variability, the reliability is low to moderate (ICC = 0.22–0.44) (23). Significant differences also exist in the GRF patterns during treadmill walking compared to overground walking, so it is unclear whether treadmills are optimal for identifying pathological gait function in neurological diseases (66–68).

In pwMS, force platforms have been used to study changes in gait initiation, postural stability, and balance associated with therapeutic interventions and disease progression (46, 69–72). Notably, Orsnes et al. (73) examined the timing of heel-contact and toe-off events in pwMS treated with baclofen (an agent used to treat spasticity in pwMS) using treadmill-embedded force platforms. The investigators observed only minimal improvements in gait and balance with treatment. A more recent study employed treadmill platforms to study both walking and jogging in minimally disabled pwMS (mean EDSS = 1.8) (25). Compared to controls, patients displayed greater step time difference between left and right feet and increased step width during both walking and jogging, but with greater change during jogging. The authors also noted that variability in the location of the CoP throughout gait cycle correlated with EDSS cerebellar scores.

Portable balance boards provide an alternative to laboratory-based force platforms. These boards use four force transducers (one on each corner of the platform) from which the CoP position can be calculated using suitable software (26). Nintendo Wii Balance Board (Nintendo, Kyoto, Japan) is the most widely tested balance board due to its low cost, portability (weighing only 3.5 kg), and wide availability. Wii Balance Board is suitable for clinical, laboratory, and home testing and demonstrates good test–retest reliability (ICC = 0.66–0.94) and construct validity when benchmarked against laboratory-grade force platforms (ICC = 0.77–0.89) (26, 27).

Wii Balance Board has been used with custom software to study postural sway in pwMS (28). Compared to laboratory force plates, Wii tended to overestimate postural sway although the test–retest reliability of the Wii has been found to be high (84%) (26–28). Castelli et al. (28) were also able to discriminate pwMS who reported fallers vs non-fallers. A case study employing Wii Balance Board noted changes in balance recorded during an exercise intervention in a single participant who had a relapse in the 6-week intervention period (29). The authors suggested that balance changes could provide a means to predict relapse onset (29). Several trials using Wii Balance Board have been undertaken and have shown potential improvements in mobility balance and QoL in pwMS (74–77), indicating that physical programs using this low cost technology could be useful for patients’ physical therapy. Overall, the cost and weight advantages of Wii, together with its high reliability and validity, make it a useful tool for assessing balance in MS in the clinic and home. Further investigations are required to identify the most useful measures that can be obtained from the device for clinical monitoring.

### Instrumented Walkways

Instrumented walkway mats are portable mats a few meters in length with sensors to identify foot contacts. GAITRite is the most commonly used instrumented walkway mat and can determine spatiotemporal measures of gait (including walking speed; step and stride lengths; base of support; step, stride, swing, stance, single support, and double support times; and toe in/out angle) with high sensitivity for detecting pathology-related changes (30, 32). Spatiotemporal outputs from GAITRite do not require skilled personnel for analysis and interpretation (78), facilitating its use in clinical settings. The GAITrite has been validated against highly advanced motion capture systems for spatiotemporal measures (33, 34) and has high test–retest reliability (ICC 0.82–0.98) (79, 80) for most gait variables in young and older healthy adults at preferred and fast walking speeds.

In pwMS, GAITrite measurement of gait variables, including time to complete, velocity, cadence and number of steps, velocity, swing time, and single support, have been shown to be comparable to the T25FW in detecting gait dysfunction (36) and correlate with cerebellar EDSS subscores (38). GAITRite is also sensitive to changes in gait in very early-stage MS in patients with minimal disability (35, 37). A key shortcoming of GAITRite for gait assessment in pwMS is the restriction of data capture to a few steps at a time. Therefore, GAITRite provides no information regarding longer-term variability on any gait measures (81), measures that have been suggested as an indicator of gait dysfunction in pwMS (14, 37).

### Advantages and Disadvantages of Non-Wearable Systems

Overall, non-wearable systems provide the most comprehensive measurements of gait kinematics available. These measurements are highly accurate, reliable, and sensitive to pathological changes, even early in the disease when clinical assessments lack sensitivity. However, these systems can be costly and are difficult to deploy in environments where everyday activities are performed (82). Low-cost marker-less optical motion capture systems such as Kinect, and portable balance boards such as Wii Balance Board, could overcome these problems, especially in clinical settings; however, as we discuss in the next section, the development of wearable technology could provide gait assessment in the community over longer time periods.

## Gait Analysis Technologies Using Wearable Sensors

Wearable sensors have been developed for detailed measurement of gait kinematics in daily life (47). They can be placed on various parts of the body (e.g., under the foot, ankle, wrist, or waist) depending on requirements (e.g., pressure measurement under foot or center of mass movement at the waist). Two of the most promising new wearable sensors used to study gait dysfunction in pwMS are pressure sensors and inertial sensors.

### Pressure Sensors

Pressure sensors are instrumented insoles placed or integrated into the shoe to measure changes in pressure between the foot and the ground. These sensors are comparable to the force platforms as they also measure the force from the ground applied to the foot, but unlike force platforms, they measure the force irrespective of its components in different directions (i.e., *x*-, *y*-, and *z*-axes) (39). Pressure sensors use plantar pressure measurements to calculate spatial-temporal gait variables, including phases of gait (e.g., stance time and swing time), and step time, length, and frequency (39, 83). There are a wide range of systems that use electromechanical sensors for plantar pressure analysis including capacitive, resistive, and piezoresistive sensors (39). When compressed, they calculate variations in applied load measuring proportional change in voltage (capacitive), conductance (resistive), or voltage (piezoresistive) (39). Arrays of sensors in configuration can measure plantar pressure in a matrix along the entire plantar surface.

The accuracy of discrete pressure sensor systems is comparable to optical motion capture (5.4% mean error) (40), external pressure calibration (ICC = 0.99), and when multiple insole pressure sensor systems are compared (ICC > 0.95) (39). In general, discrete and matrix pressure sensor insoles have good to excellent reliability for pressure measurements within and between trials (ICC = 0.80–0.99) (39, 84). However, as gait speed affects plantar pressure, it is recommended that gait speeds are controlled when collecting gait data with pressure sensors (84).

Three studies have used pressure sensor technology to study gait dysfunction in pwMS. One study used discrete pressure sensor insoles combined with mobile technology that included a hand held mobile device, to assess plantar pressure and step timing and observed greater plantar pressure in stance phase and greater variability in step timing in pwMS compared to controls (41). Two related studies assessed gait in early-stage MS (4) and changes in gait over the subsequent 12 months (52). Cross-sectionally, pwMS patients with pyramidal signs displayed increased double limb support and decreased walking speed and stride length compared to those with no pyramidal signs (4). Longitudinally, pwMS exhibited a decline in gait performance over 12 months in the absence of EDSS change (52). These results demonstrate that pressure sensors have the sensitivity to detect gait dysfunction in patients with no or minimal clinical disability.

### Inertial Sensors

**Figure 1:**
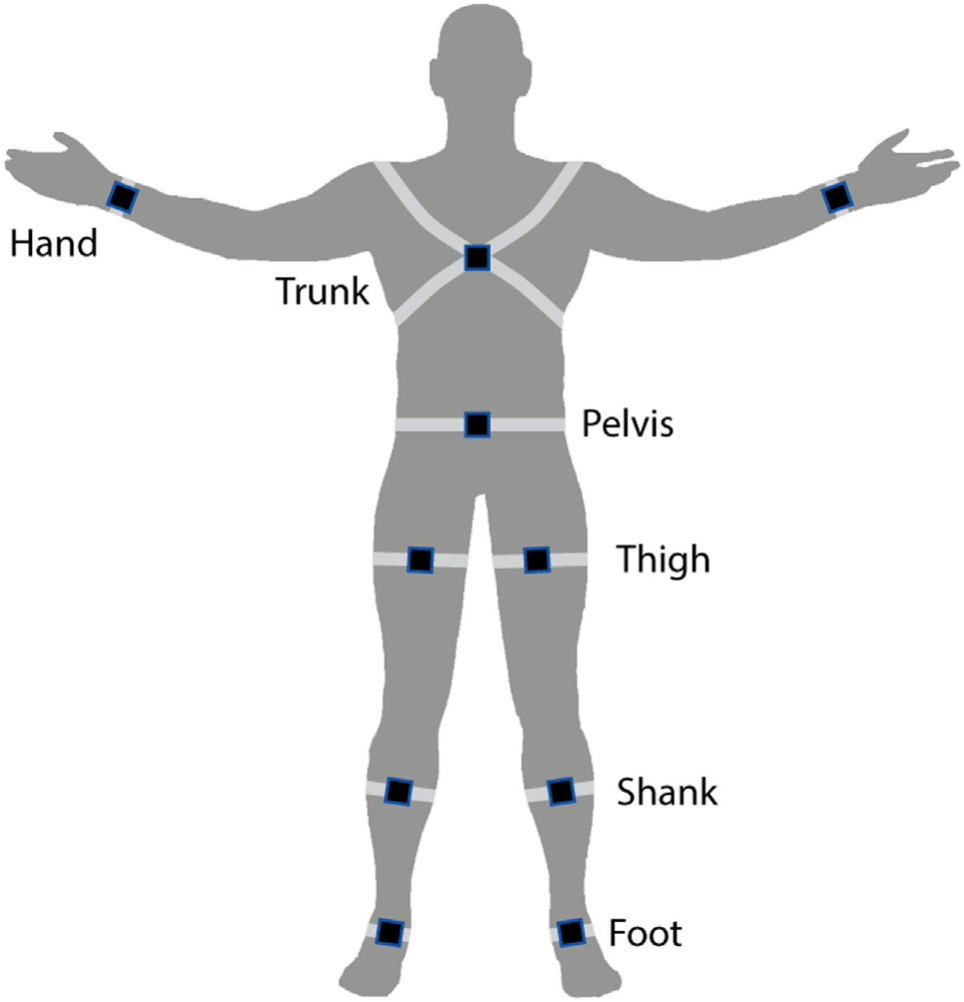
Illustration of common inertial sensor placements on the body.

Inertial sensors measure an object’s acceleration and can also be used to report velocity, orientation, and gravitational forces. Inertial sensors are the most widely used type of wearable systems for gait and balance analysis and have been validated in healthy volunteers and in groups with motor impairment (85–87). The most promising inertial sensors for 3D gait analysis consist of a combination of tri-axial accelerometer, tri-axial gyroscope, and tri-axial magnetometer. Tri-axial sensors can capture spatiotemporal (e.g., swing time and cadence) and 3D kinematic data including joint and segment angles. Similar to the pressure sensors, inertial sensors can be integrated into insoles making them highly suitable for gait analysis. However, they can also be attached to other parts of the body such as on a belt or the wrist as illustrated in Figure 1. Additionally, technology is being developed for inertial sensor data collection, storage and/or transmission with smart devices such as phones and watches (88–90).

Trunk- or shank-placed inertial sensors have been used to study gait dysfunction in pwMS, commonly during the TUG test (termed “instrumented TUG”) (14, 42, 44, 46, 91, 92). Spain et al. (91) reported increased sway acceleration during quiet stance with eyes closed and increased trunk motion during instrumented TUG in pwMS with normal walking speed. In a follow-up longitudinal study (14), the authors assessed changes in gait and balance over 18 months, demonstrating no worsening of balance and objective gait measures (sway and gait velocity, respectively), but differentiation of mild MS (average EDSS = 2.2), moderate MS (average EDSS = 4.3), and control groups based on gait velocity, trunk motion, sway range, and sway area. Variability in sway area, sway range, and trunk motion over time were significantly different between all three groups. Similarly, Solomon et al. (93) found that inertial sensor data differentiated pwMS and no clinical gait dysfunction from controls using measures of postural sway (mediolateral sway path length and mediolateral sway range). Importantly, inertial sensors during TUG appear to be quite reproducible (ICC > 0.85 for all trunk and shank recordings from pwMS tested over two sessions), and some variables (stride velocity, cadence, and cycle time) correlate significantly with EDSS and number of recent falls (92).

### Advantages and Disadvantages of Wearable Systems

The great advantage of wearable sensors is the ability to measure gait in an individual patient’s everyday environment for extended periods of time. These systems now employ small wireless sensors that can remotely send signals to the laboratory or clinic. Connectivity between wearable systems and ubiquitous smart phones and watches could further improve the usability of these devices. Importantly, the cost of wearable sensors is generally lower than non-wearable systems making analyses on large numbers of patients feasible. Finally, wearable systems actively engage the patient in both assessment and rehabilitation and could reduce clinic visits by providing more real time information to the patient and treating clinician (94).

Wearable systems also have certain disadvantages. First, wearable sensors can generally measure a smaller number of gait variables than non-wearable laboratory systems. Therefore, early studies of wearables should involve benchmarking and validation against these more comprehensive systems. Second, the placement of the sensors on body parts could hinder daily activities, though this could be improved with integration of sensors into clothing, smartphones, and watches. Third, algorithms used to measure speed and distance with wearable systems can lead to amplification of measurement error (95). Indeed, the algorithms required to calculate gait variables, which in some cases require technical personnel to implement, are currently a barrier to clinical application. However, algorithm development is an active area of research and clinician and patient interfaces continue to improve (96). Finally, the use of wearable sensors by patients themselves in uncontrolled everyday environments can make them more susceptible to signal noise (e.g., magnetic or vibration interference), leading to incorrect data and inadequate durations of recording when out of the clinic (97).

## Conclusion and Future Directions

Escalating treatment in response to changing disease state in early MS can substantially affect outcomes, and identifying change in disease state throughout the course of MS is essential for optimal treatment (6). Current clinical and performance tests (EDSS and T25FW) for assessing gait function in pwMS are adequate for identifying advanced gait dysfunction, but fail to detect early subtle gait dysfunction or progression. In contrast, advanced motion analysis using wearable and non-wearable systems can overcome these problems. Laboratory-based systems offer the greatest sensitivity and are reliable over a wide spectrum of measures; however, these are costly, time- and space-intensive, and require technical skills for operation. Portable (i.e., Kinect, Wii Balance Board, and GAITRite) and wearable sensors offer less expensive alternatives for reliably measuring gait and balance variables and can be applied both in and out of the clinic. An example clinical application is balance training interventions for preventing falls (98, 99) that could be deployed and assessed using simple balance board technologies.

Future developments in portable and wearable systems will, in our opinion, allow these technologies to be used for monitoring and predicting disability in real-world environments. The feasibility of using wearable sensors has already been demonstrated for monitoring gait characteristics related to fall risk and symptoms in small groups of older adults (100) and people with Parkinson’s disease (101). Further studies are needed to investigate: (a) the gait characteristics that predict change in symptoms such as falls, relapses, or disability progression, and (b) the feasibility and utility of continuous monitoring of gait and balance in pwMS.

## Author Contributions

CS, FB, LL, and SK made substantial contributions to the conception and design of this work, drafting the work and revising it critically for important intellectual content; gave final approval of the version to be published; and agree to be accountable for all aspects of the work in ensuring that questions related to the accuracy or integrity of any part of the work are appropriately investigated and resolved. AW, MG, MS, BM, FK and TK made substantial contributions to the conception and design of this work; revising it critically for important intellectual content; gave final approval of the version to be published; and agree to be accountable for all aspects of the work in ensuring that questions related to the accuracy or integrity of any part of the work are appropriately investigated and resolved.

## Conflict of Interest Statement

No author of this manuscript has received financial support or other benefits from commercial sources for the work reported in the manuscript, or has any other financial interests that could create any potential conflict of interest or the appearance of a conflict of interest with regard to the work. The reviewer WS-M and handling editor declared their shared affiliation.
